# S3 Guideline Allergy Prevention*

**DOI:** 10.5414/ALX02303E

**Published:** 2022-03-04

**Authors:** Matthias V. Kopp, Cathleen Muche-Borowski, Michael Abou-Dakn, Birgit Ahrens, Kirsten Beyer, Katharina Blümchen, Petra Bubel, Adam Chaker, Monika Cremer, Regina Ensenauer, Michael Gerstlauer, Uwe Gieler, Inga-Marie Hübner, Fritz Horak, Ludger Klimek, Berthold V. Koletzko, Sybille Koletzko, Susanne Lau, Thomas Lob-Corzilius, Katja Nemat, Eva M.J. Peters, Antonio Pizzulli, Imke Reese, Claudia Rolinck-Werninghaus, Elien Rouw, Bianca Schaub, Sebastian Schmidt, Jens-Oliver Steiß, Anne Kathrin Striegel, Zsolt Szépfalusi, Dietmar Schlembach, Thomas Spindler, Christian Taube, Valérie Trendelenburg, Regina Treudler, Ulrich Umpfenbach, Christian Vogelberg, Martin Wagenmann, Anke Weißenborn, Thomas Werfel, Margitta Worm, Helmut Sitter, Eckard Hamelmann

**Affiliations:** 1 Airway Research Center North, University of Lübeck, Member of Deutsches Zentrum für Lungenforschung, Universitätsklinik für Kinderheilkunde, Inselspital, Bern, Switzerland,; 2 Institut für Allgemeinmedizin, University Medical Center Hamburg-Eppendorf, Hamburg, Germany,; 3 Clinic for Gynecology and Obstetrics, St. Joseph-Krankenhaus Berlin-Tempelhof, Germany,; 4 Children’s Hospital, University Hospital Frankfurt, Germany,; 5 Department of Pediatric Respiratory Medicine, Immunology and Critical Care Medicine, Charité Universitätsmedizin Berlin, Germany,; 6 HNO-Facharztpraxis, Eisleben, Germany,; 7 HNO-Klinik, Klinikum rechts der Isar, Technical University of Munich, Munich, Germany,; 8 Ökotrophologin, Journalistin, Idstein/Taunus, Germany,; 9 Institut für Kinderernährung, Max Rubner-Institut, Karlsruhe, Germany,; 10 Kinderklinik, Universitätsklinikum Augsburg, Germany,; 11 Klinik für Psychosomatik und Psychotherapie des UKGM, Universitätsklinik, Giessen, Germany,; 12 Arbeitsgemeinschaft Dermatologiche Prävention e.V., Hamburg, Germany,; 13 Allergiezentrum Wien West, Vienna, Austria,; 14 Zentrum für Rhinologie und Allergologie, Wiesbaden, Germany,; 15 Integriertes Sozialpädiatrisches Zentrum, Dr. von Haunerschen Kinderspital, LMU Klinikum der Universität München, Munich, Germany,; 16 Abteilung für Stoffwechsel und Ernährung, Dr. von Haunersches Kinderspital, LMU Klinikum der Universität München, Munich, Germany,; 17 Kinder- und Jugendmedizin, Christliches Kinderhospital Osnabrück, Germany,; 18 Kinderzentrum Dresden-Friedrichstadt, Dresden, Germany,; 19 Schwerpunktpraxis für Allergologie und Lungenheilkunde im Kinder- und Jugendalter, Berlin, Germany,; 20 Ernährungsberatung und -therapie mit Schwerpunkt Allergologie, Munich, Germany,; 21 Praxis für Kinder- und Jugendmedizin, Teltow, Germany,; 22 Kinderarztpraxis, Bühl, Germany,; 23 Asthma- und Allergieambulanz, Dr. von Haunersches Kinderspital, LMU Klinikum der Universität, Munich, Germany,; 24 Allgemeine Pädiatrie, Klinik und Poliklinik für Kinder- und Jugendmedizin, Universitätsmedizin Greifswald, Greifswald, Germany,; 25 Facharztpraxis für Kinder- und Jugendmedizin, Fulda, Germany,; 26 Kinder- und Jugendmedizin, Universitätsklinikum, Cologne, Germany,; 27 Universitätsklinik für Kinder- und Jugendheilkunde, Medizinische Universität Wien, Vienna, Austria,; 28 Klinik für Geburtsmedizin, Vivantes Klinikum Neukölln, Berlin, Germany,; 29 Hochgebirgsklinik Davos, Switzerland,; 30 Klinik für Pneumologie, Ruhrlandklinik, Westdeutsches Lungenzentrum am Universitätsklinikum, Essen, Germany,; 31 Klinik für Dermatologie, Venerologie und Allergologie, Leipziger Allergie-Centrum LICA – CAC, Universitätsmedizin, Leipzig, Germany,; 32 Praxis für Kinder- und Jugendmedizin, Dülken, Germany,; 33 Klinik und Poliklinik für Kinder- und Jugendmedizin, Universitätsklinikum Carl Gustav Carus an der Technischen Universität, Dresden, Germany,; 34 HNO-Klinik, Universitätsklinikum Düsseldorf, Düsseldorf, Germany,; 35 German Federal Institute for Risk Assessment, Berlin, Germany,; 36 Klinik für Dermatologie, Allergologie und Venerologie, Medizinische Hochschule Hannover, Hannover, Germany,; 37 Klinik für Dermatologie, Allergologie und Venerologie, Campus Charité Mitte, Universitätsmedizin Berlin, Berlin, Germany,; 38 Institut für Chirurgische Forschung, Philipps-Universität, Marburg, Germany, and; 39 Kinder-Zentrum Bethel, Evangelisches Klinikum Bethel, Universitätsklinik für Kinder- und Jugendmedizin, Universitätsklinikum OWL, Universität Bielefeld, Bielefeld, Germany

**Keywords:** allergy, evidence, S3 Guideline, primary prevention, revision

## Abstract

Background: The persistently high prevalence of allergic diseases in Western industrial nations and the limited possibilities of causal therapy make evidence-based recommendations for primary prevention necessary. Methods: The recommendations of the S3 Guideline Allergy Prevention, published in its last version in 2014, were revised and consented on the basis of a current systematic literature search. The evidence search was conducted for the period 06/2013 – 11/2020 in the electronic databases Cochrane and MEDLINE, as well as in the reference lists of current reviews and through references from experts. The literature found was screened in two filtering processes, first by title and abstract, and the remaining papers were screened in the full text for relevance. The studies included were sorted by level of evidence, and the study quality was indicated in terms of potential bias (low/high). The revised recommendations were formally agreed and consented upon with the participation of representatives of the relevant professional societies and (self-help) organizations (nominal group process). Of 5,681 hits, 286 studies were included and assessed. Results: Recommendations on maternal nutrition during pregnancy and breastfeeding as well as on infant nutrition in the first months of life again play an important role in the updated guideline: Many of the previous recommendations were confirmed by the current data. It was specified that breastfeeding should be exclusive for the first 4 – 6 months after birth, if possible, and that breastfeeding should continue with the introduction of complementary foods. A new recommendation is that supplementary feeding of cow’s milk-based infant formula should be avoided in the first days of life if the mother wishes to breastfeed. Furthermore, it was found that the evidence for a clear recommendation for hydrolyzed infant formula in non-breastfed infants at risk of atopic diseases is currently insufficient. It is therefore recommended to check whether an infant formula with proven efficacy, demonstrated in allergy prevention studies, is available until the introduction of complementary feeding. Finally, based on the EAACI guideline, recommendations were made for the prevention of hen’s egg allergy by introducing and regularly giving thoroughly heated (e.g., baked or hard-boiled) but not “raw” hen’s egg (also no scrambled egg) with the complementary food. The recommendation to introduce peanut in complementary feeding was formulated cautiously for the German-speaking countries: In families with regular peanut consumption, the regular administration of peanut-containing foods in age-appropriate form (e.g., peanut butter) with the complementary diet can be considered for the primary prevention of peanut allergy in infants with atopic dermatitis (AD). Before introduction, a clinically relevant peanut allergy must be ruled out, especially in infants with moderate to severe AD. There is still insufficient evidence for an allergy-preventive efficacy of prebiotics or probiotics, vitamin D, or other vitamins in the form of supplements so that recommendations against their supplementation were adopted for the first time in the current guideline. Biodiversity plays an important role in the development of immunological tolerance to environmental and food allergens: there is clear evidence that growing up on a farm is associated with a lower risk of developing asthma and allergic diseases. This is associated with early non-specific immune stimulation due to, among other things, the greater microbial biodiversity of house dust in this habitat. This aspect is also reflected in the recommendations on animal husbandry, on which a differentiated statement was made: In families without a recognizable increased allergy risk, pet keeping with cats or dogs should not generally be restricted. Families with an increased allergy risk or with children with already existing AD should not acquire a new cat – in contrast, however, dog ownership should not be discouraged. Interventions to reduce exposure to dust mite allergens in the home, such as the use of mite allergen-proof mattress covers (“encasings”), should be restricted to patients with already proven specific sensitization against house dust mite allergen. Children born by caesarean section have a slightly increased risk of asthma – this should be taken into account when advising on mode of delivery outside of emergency situations. Recent work also supports the recommendations on air pollutants: Active and passive exposure to tobacco smoke increase the risk of allergies, especially asthma, and should therefore be avoided. Exposure to nitrogen oxides, ozone, and small particles (PM 2.5) is associated with an increased risk, especially for asthma. Therefore, exposure to emissions of nitrogen oxides, ozone, and small particles (PM 2.5) should be kept low. The authors of this guideline are unanimously in favor of enacting appropriate regulations to minimize these air pollutants. There is no evidence that vaccinations increase the risk of allergies, but conversely there is evidence that vaccinations can reduce the risk of allergies. All children, including children at risk, should be vaccinated according to the current recommendations of the national public health institutes, also for allergy prevention. Conclusion: The consensus of recommendations in this guideline is based on an extensive evidence base. The update of the guideline enables evidence-based and up-to-date recommendations for the prevention of allergic diseases including asthma and atopic dermatitis.

*As of November 28, 2021

**Jointly and equally coordinated this guideline

AWMF Registry number 061-016

## Materials and methods 

The procedure for creating the guideline is described in detail in the guideline report (Supplemental material). 

### Objectives 

The primary targets of this guideline are the most important atopic diseases: atopic eczema, food allergy, allergic rhinoconjunctivitis, and (allergic) asthma. The guideline mainly refers to primary prevention measures and is based on the following definitions, modified for the field of allergies: 

Primary prevention includes, on the one hand, the elimination or reduction of (partial) causes that are important for the development of the disease, including changes in causal or predisposing environmental and workplace-related factors, and, on the other hand, the increasing tolerance of individuals. Primary prevention is mainly relevant for risk groups (genetic predisposition) but is also aimed at the general population and includes aspects of allergy-specific health promotion. 

The target groups of secondary prevention are people with early signs of disease (e.g., bronchial or nasal hyperreactivity with proven sensitization) and sensitized but still asymptomatic people. The objectives of secondary prevention are to avoid disease manifestation and change in symptoms. The measures of secondary prevention include the avoidance of clinically relevant allergens and toxic-irritative substances, advice and, in the case of people with early signs of disease, pharmaco-prophylaxis and allergen-specific immunotherapy (desensitization) if appropriate. 

Following this definition, the measures in the recommendation algorithm are subdivided, where applicable, into genetically predisposed and non-predisposed people. Studies on patients with manifest disease, including those aimed at preventing a second disease, were not taken into account. The target population are people, particularly children, with and without genetic predisposition for atopic diseases. Children with a genetic predisposition (“children with an increased risk of atopic diseases”) are defined as having at least one parent or sibling suffering from one of the atopic diseases mentioned (bronchial asthma, allergic rhinitis, atopic dermatitis, food allergy). Thus, in addition to the general population, young families, couples who wish to have children, or pregnant women as well as people with a relevant family history can be considered as target groups. 

### Target group 

The guideline is aimed at medical and non-medical specialists who care for the people defined as the target population as part of their work. 

User target group/addressees: Users and multipliers of the guideline contents are all medical and non-medical associations and groups of people involved in preventive measures and in particular in allergy prevention. In addition to representatives of the relevant groups of specialists, professionals, and affected persons, doctors from all specialist groups, in particular pediatricians, dermatologists, ENT doctors, and pulmonologists and allergologists, as well as self-help organizations and those otherwise affected, are considered addressees. 

### Search for evidence 

The search for evidence was carried out for the period 06/2013 – 11/2020 in the electronic databases Cochrane and MEDLINE, as well as in the reference lists of current reviews. Additional studies were identified by the experts involved. The literature found was checked for relevance in two filtering processes, initially according to title and abstract, and the remaining papers in full text. The studies included afterwards were classified according to the level of evidence, and the study quality was indicated according to the of the risk of bias (low/high). The revised recommendations were formally coordinated and consented to with the participation of representatives from the relevant specialist societies and (self-help) organizations (nominal group process). 

Of 5,681 search hits, 286 studies were included and evaluated. The procedure for evidence search and evaluation is described in detail in the guideline report. 

### Drafting of the guideline 

Based on the found and evaluated studies, a proposal for the revised recommendations for prevention was circulated in preparatory meetings in different working groups. Suggestions for amendments and revision were discussed and, if necessary, incorporated. In addition, background texts on the individual topics were prepared in the working groups, which were then merged and harmonized by the guideline coordinators. 

### Consensus 

All persons who had participated in the development and approval of the 2014 version of the guideline were invited to the consensus group. In addition, representatives from other specialist societies were appointed upon proposal. The recommendations were approved by the consensus group. A maximum of two representatives with one common voting right were allowed per organization. The nominal group process technique was used as the formal consent process. A total of three online-based meetings took place. The process was strictly structured (see below). 

The web-based consensus meetings took place in November 2020, December 2020, and March 2021 and were chaired by PD Dr. H. Sitter (University of Marburg and AWMF). 

The process of the nominal group technique was as follows: 1) Presentation of the statements to be consented. 2) Each participant prepares comments and discussion requests on the given statements. 3) The chair requests the comments from each participant in turn, and similar comments are summarized. 4) For each point, a vote is taken as to who would like to discuss this point. 5) The topics are ranked according to these votes. (6) In a joint group, the individual members, one after the other, give their opinions on the individual discussion points. 7) After several rounds, the participants finally agree on a certain formulation by voting or ranking. 8) Steps 1 – 6 are repeated for each statement under discussion. 

Based on the evidence, the recommendations that have been consented to are referred to as proofs or indications. This terminology is based on the methods formulated by IQWIG (Institut für Qualität und Wirtschaftlichkeit im Gesundheitswesen (Institute for Quality and Efficiency in Health Care)). In “General Methods 6.0” it says, among other things: 

„Medical interventions are compared with other interventions, sham interventions (e.g. placebo), or no intervention in respect of their effects on defined patient-relevant outcomes, and their (added) benefit and harm are described in summary. For this purpose, on the basis of the analysis of the scientific data available, for each predefined patient-relevant outcome separately a conclusion on the evidence base of the (added) benefit and harm is drawn in 4 levels with regard to the respective certainty of the conclusion: The data provide either “proof” (highest certainty of conclusions), an “indication” (medium certainty of conclusions), a “hint” (weakest certainty of conclusions) in respect of the benefit or harm of an intervention, or none of these 3 situations applies. The latter is the case if no data are available or the data available do not allow any of the other 3 conclusions to be drawn.“

The recommendations were individually adopted by the consensus group with a class of recommendation (A, B, C, or D), which is added to the respective recommendation in brackets. The classes of recommendation can be assigned in a formalized form based on the level of evidence (see guideline report). In justified cases, however, deviating classes of recommendation could also be adopted as part of the consent process. On topics for which no prevention recommendations could be derived, only statements were formulated. 

All recommendations could be adopted by strong consensus (> 95% agreement of the participants), by consensus (> 75 – 95% agreement), or with a majority agreement (> 50 – 75% agreement); the levels of consensus are indicated with the recommendations. 

## Results 

The consented recommendations for the primary prevention of bronchial asthma, allergic rhinitis, food allergy, and atopic eczema apply to people at risk and those not at risk, unless explicitly stated otherwise, and are as follows: [Table Table1][Table Table2][Table Table3][Table Table4][Table Table5][Table Table6][Table Table7][Table Table8][Table Table9][Table Table10][Table Table11][Table Table12][Table Table13], [Table Table14][Table Table15][Table Table16][Table Table17][Table Table18][Table Table19][Table Table20][Table Table21]

## Discussion 

### Nutrition 

During pregnancy and lactation, a balanced and diverse diet covering all nutritional requirements is recommended. Although the topic of “dietary diversity during pregnancy” has been discussed to lower the risk of allergic diseases in childhood in the past [1], recent studies failed to show any or only a limited protective effect of a diverse diet during pregnancy and breastfeeding: While indications of a beneficial effect of a greater variety of fermented foods on atopic dermatitis were seen in a case-control study [2], a birth cohort study showed no relationship between a balanced diet and the manifestation of allergic diseases in children between 3 and 10 years [3]. In another cohort study, the highest consumption of cabbage and other vegetables rich in folate in the first trimester, compared to the lowest consumption, was associated with a significantly lower risk of wheezing in children aged 2 years. However, this association could no longer be seen for the second and third trimester [4]. Earlier indications of a protective effect of a Mediterranean diet during pregnancy on the development of allergic diseases in childhood (Update 2014) were not supported by the results of a population-based birth cohort [5]. 

Previous indications (update 2014) of a preventive effect of fish consumption during pregnancy and breastfeeding on allergic respiratory diseases in children could neither be confirmed in recent birth cohorts [6, 7, 8, 9, 10] nor in a pooled analysis of various cohorts with a total of 60,774 mother-child pairs [11]. Furthermore, no protective effect of fish consumption on atopic dermatitis or other allergic diseases can be derived from the current data [7, 8, 9, 10]. 

Two prospective cohort studies confirm earlier indications of an association between higher total omega-6 PUFA (polyunsaturated fatty acids) levels in the mother’s blood during pregnancy and a higher incidence of atopic dermatitis in children [7, 12], especially in children with a relevant family history [12]. Linoleic acid, an omega-6 fatty acid, is found in many vegetable and seed oils. The positive association observed by Rucci et al. [7] is largely attributed by the authors to high maternal linoleic acid levels. In contrast, there are inconsistent results regarding the association between total omega-6 PUFA levels in the mother’s blood during pregnancy and asthma in the child: While an inverse association with asthma onset at 6 years of age was observed in a prospective cohort study of 4,976 mother-child pairs by Rucci et al. [7], higher maternal levels were positively associated with childhood asthma in 4- to 6-year-olds in another prospective study including 1,019 mother-child pairs. The association was shown to be more evident when the mother herself suffered from asthma [13]. 

Observational studies in recent years indicated possible protective effects of milk and milk products, including fermented milk products such as yogurt, in particular for atopic dermatitis [2, 14] but also for allergic respiratory diseases [14, 15]. A study that analyzed, among other things, fatty acids derived from ruminants in the mother’s plasma during pregnancy suggests an inverse relationship between the risk of developing atopic dermatitis in the first 14 months of life and the maternal plasma level of vaccenic acid, which is typical for ruminants [16]. 

The body of evidence is insufficient for specific recommendations for targeted consumption of individual foods, but still supports a balanced, varied, and nutritious diet during pregnancy and breastfeeding, which among other things includes vegetables, milk, and dairy products, including fermented milk products (such as yogurt), fruits, nuts, eggs, and fish. 

Dietary restriction has not been recommended anymore for a long time. There are no current studies on this topic. The EAACI guideline on the prevention of food allergies recommends no avoidance of potential food allergens during pregnancy and breastfeeding in order to prevent food allergies in infants and children [17]. 

### Breastfeeding 

Breastfeeding has many benefits for both mother and child, even if the infant is only partially breastfed. Mothers should be provided with comprehensive information and support to enable them to breastfeed their child. 

Even if breast milk should be ideal for reducing the risk of allergic diseases due to the numerous immunologically active substances and favorable effects on the child’s microbiome, existing data on this issue are inconsistent [18]. This is, among other things, due to the following reasons: breastfeeding cannot be studied in randomized trials for ethical reasons; existing studies are difficult to compare due to their different designs and their different definitions of breastfeeding; the studies used different endpoint parameters. For example, many studies do not really distinguish between “exclusive breastfeeding” and “predominant breastfeeding”. According to the WHO definition, “exclusive breastfeeding” is the exclusive use of breast milk, while “predominant breastfeeding” means that breast milk is the predominant source of food, but the infant may also receive liquids, food supplements, and medication (http://apps.who.int/iris/bitstream/handle/ 10665/43895/ 9789241596664_eng.pdf; jsessionid=6418566B6D27 863F9FE4BD72 AF65FC26?sequence=1). In addition, mothers who exclusively breastfeed differ in many parameters that are important for the development of allergies from mothers who feed infant formula: age, smoking during and after pregnancy, socio-economic status, level of education, pet keeping, introduction of complementary food [19, 20]. A further complicating factor is that the composition of breast milk is complex and can vary greatly from one individual to another with regard to many substances that may be relevant to allergy prevention [18]. 

Most current studies show no indication of protective effects [21, 22, 23, 24, 25, 26] – neither with exclusive nor with any form of breastfeeding. The endpoints were allergic respiratory disease, especially asthma, and in some studies also atopic dermatitis. However, the effect of reverse causation should be kept in mind: children at high risk of atopic dermatitis may be breastfed for a longer time. In one study, the development of eczema was slightly inversely associated with breastfeeding, especially with breastfeeding for more than 6 months or with exclusive breastfeeding for 4 months [27]. The follow-up results of the GINI intervention cohort also suggest a protective effect of breastfeeding – but only in infants with an increased allergy risk [28]. After adjustment for possible confounding factors, there was a significantly reduced cumulative incidence of eczema in children and adolescents who had received exclusively breast milk for the first 4 months of their lives compared to those who had received cow’s milk-based infant formulas. 

Some studies also suggest a protective effect on the development of asthma or wheezing with exclusive breastfeeding in the first 3 months [27, 29], 6 months [30] as well as with longer breastfeeding [31, 32, 33, 34]. 

Despite the contradicting data, the recommendation in Germany to exclusively breastfeed in the first 4 – 6 months is supported. Breastfeeding should be continued when complementary foods are introduced. 

Temporary feeding of infant formulas in the first days of life is still widespread in many industrialized countries and also in Germany [35]. In accordance with the EAACI guideline on the prevention of food allergies, the recommendation to avoid temporary feeding of cow’s milk-based formula in the first days of life has been adopted [17]. The recommendation is based on a study that has shown that the administration of an amino acid formula compared to a cow’s milk-based infant formula was associated with a significant reduction of the risk of sensitization and allergy to cow’s milk in early childhood [36]. Earlier data suggest that the administration of an extensively hydrolyzed therapeutic formula was also associated with a comparable risk reduction [37]. Therefore, if a temporary supplementation with formula is medically indicated in the first days of life, this should be done with an extensively hydrolyzed therapeutic formula or amino acid formula. Whether partially hydrolyzed formulas are also effective for allergy prevention cannot be deduced from the currently available data. 

### Breast milk substitutes and cow’s milk substitutes in children at risk 

The current follow-up of the randomized controlled intervention study GINI confirms a sustained risk-reducing effect of specific hydrolyzed formula for atopic dermatitis even after 15 years [38]. In contrast, in a French birth cohort including 11,720 children, the use of partially hydrolyzed infant formula was not associated with a lower risk of atopic diseases (eczema, respiratory symptoms, or food allergy); but was actually associated with an increased risk of wheezing at 1 year of age in infants at increased risk of atopic disease [39]. However, the risk analysis included only the type of feeding during the first two months of life. A calculated increased risk of eczema at 1 year of age disappeared after excluding infants in whom allergy symptoms were reported at 2 months of age. It also must be taken into account that observational studies cannot provide the same reliable basis for recommendations results from randomized controlled trials provide [40]. 

With regard to the recommendation in favor of hydrolyzed infant formula, the following should be considered:

- The evidence on a preventive use has to be considered in a product-specific way; an overall assessment is hardly possible due to various factors *(these include, for example: the choice of protein hydrolysate per se (protein source, hydrolysis processes, peptide sizes), the evident heterogeneity in the study designs including duration of the study/intervention, study population, group sizes, endpoints, but also the influence of the industry, etc.).* As a result, there are controversial national and international discussions regarding the effectiveness of partially or extensively hydrolyzed infant formulas for the purpose of allergy prevention in general or with regard to a *(focused)* prevention of atopic dermatitis, cow’s milk allergy, asthma, and/or hay fever in young children and older children [41, 42, 43, 44].
- The hydrolyzed infant formulas studied in previous studies are no longer available on the German market or are no longer available in their original composition.
- The current requirements by EFSA as layed down for manufacturers in the Commission Delegated Regulation (EU) 2021/572 of January 20, 2021 amending Delegated Regulation (EU) 2016/127 also are to apply to infant formula and follow-on formula based on protein hydrolysates from February 22, 2022. These include the requirement for proof of effectiveness derived from clinical studies that demonstrate “if and to what extent a particular formula reduces the risk of developing short and long-term clinical manifestations of allergy in at-risk-infants who are not breast-fed.”... “In addition if, after the assessment by the Authority, it is demonstrated that a specific infant formula manufactured from protein hydrolysates reduces the risk of developing allergy to milk proteins, further consideration will be given to how to adequately inform parents and caregivers about that property of the product.” (Commission Delegated Regulation (EU) 2016/127 on infant formula and follow-on formula and the nutrition of infants and young children).
- In a recent opinion, EFSA has assessed an infant formula manufactured from the hydrolyzed whey protein currently authorized by Delegated Regulation (EU) 2016/127 with regard to reducing the risk of atopic dermatitis in infants with an increased risk and concluded that no such risk-reducing effect can be derived from the presented data [45].

In view of the aspects mentioned, infants who are not or only partially breastfed should be given an infant formula. For children with an increased risk of atopic disease, it should be checked whether an infant formula with proven effectiveness, demonstrated in allergy prevention studies, is available until complementary food is introduced. 

There is no proof of an allergy-preventive effect of animal milk such as goat’s, sheep’s, or mare’s milk, including infant formula made from goat’s milk. Also, the use of soy-based infant formula cannot be recommended for the purpose of allergy prevention [46]. Moreover, there are health concerns related to soy-based infant formula (high content of phytochemicals with a weak estrogenic effect and phytates with possible disadvantages for nutrient absorption) [47, 48], which, however, are controversially discussed [49]. The use of soy-based infant formula is recommended by the German Society of Pediatrics DGKJ (Deutsche Gesellschaft für Kinder und Jugendheilkunde) only for special indications (galactosemia or ethical or cultural reasons) [50]. The DGKJ recommendation does not include a statement on the use of calcium-fortified soy products (soy drink, yoghurt, etc.) or on cereal-based and other plant-based drinks as part of the complementary food. Drinks based on cereals and other plants, even with calcium fortification, are to be viewed critically due to their low fat and protein quantity and quality, and should therefore not be used as a substitute for cow’s milk [52]. 

### Introduction of complementary foods 

In Germany it is recommended to start feeding complementary food from the beginning of the 5^th^, at the earliest, to the beginning of the 7^th^ month of life, at the latest, depending on the readiness of the baby. 

While the 2014 update mainly indicated that the introduction of fish has a protective effect against the development of allergic diseases, recent publications focus more on the diversity of the diet: The current data confirm earlier indications (update 2014) of a positive effect of fish consumption [6, 53, 54, 55], especially on allergic asthma/wheezing. In addition, three cohort studies were able to show that a wide variety of complementary foods was associated with a lower incidence of atopic dermatitis and/or allergic respiratory diseases [56, 57, 58]. Thereby, the development of atopic eczema was significantly less frequent in children who had received complementary food at the age of 4 or 5 months compared to those who were exclusively breastfed – even when children with and without allergy risk were considered separately [56]. Nwaru et al. [57] did not observe any association between food diversity and atopic diseases at the age of 3 and 4 months. From the age of 6 months, on the other hand, a lower variety of complementary foods was associated with an increased risk of atopic diseases. An earlier introduction of fish (< 29 weeks) was also associated with a lower risk of developing asthma compared to a later introduction (≥ 43 weeks) with a lower risk of developing asthma [53]. In a recent EAACI position paper [1], the authors “recommend that infants of any risk category for allergic disease should have a diverse diet, given no evidence of harm and some potential association of benefit in the prevention of particular allergic outcomes.” 

Earlier indications from the 2014 update on a protective effect of a Mediterranean diet, as well as of omega-3 fatty acids and milk can possibly be explained by more recent and future study results on butyrate and the microbiome [59, 60]. Butyrate is a short-chain fatty acid that is produced by specific intestinal bacteria and modulates the activity of immune cells. Roduit et al. [61] were able to show in a cohort study that the consumption of vegetables, fish, and yogurt in the first year of life was associated with an increased concentration of butyrate in the stool and inversely associated with allergic respiratory diseases between the ages of 3 and 6 years. However, two other cohort studies did not observe an association between the consumption of vegetables and fruits and the development of asthma [53, 55]. In contrast, the consumption of dairy products was inversely associated with the risk of atopic asthma in one of the two cohorts [55]. 

A protective effect of yogurt in the first year of life on the development of atopic dermatitis was suggested in an observational study [62] and in an evaluation of the consumption of yogurt by infants during the complementary feeding period, which was determined in a randomized-controlled administration of probiotics to women from pregnancy through to the breastfeeding period [63, 64]. In the studies by Crane et al. [63] and Du Toit et al. [64], the inverse association between the administration of yogurt and the risk of atopic dermatitis was significantly more pronounced when yogurt was introduced in the first 6 months of life and given regularly. 

There is no proof of a preventive effect of dietary restriction by avoiding potent food allergens in the first year of life. Therefore, no restriction should be made. 

With reference to the EAACI guideline on the prevention of food allergies, which is based on a systematic review of the literature [43], the recommendation to introduce hen’s eggs in a sufficiently heated form (e.g., hard-boiled) is adopted [17]. Both underlying studies used hard-boiled egg, but it is assumed that baked hen’s egg has a similar effect. This includes, among other things, well-baked egg-containing baked goods (such as hard biscuits, specialty breads and rolls as well as muffins and cakes). Once successfully introduced, hen’s eggs should be given regularly in a sufficiently heated form. Since the introduction of unheated or insufficiently heated eggs was not associated with any benefit but with risks of severe allergic reactions, the administration of raw (not fully heated) hen’s eggs is not recommended for the prevention of egg allergy. This also includes scrambled eggs, soft-boiled eggs as well as marshmallow treats and macarons. 

The recommendation made for countries with a high prevalence of peanut allergy to specifically introduce peanut products is not adopted since Germany cannot be classified as such a country. Only in families with regular peanut consumption and presence of atopic dermatitis in the infant, a targeted introduction of peanut products in an age-appropriate form (because of the risk of aspiration, not whole peanuts or pieces), may be considered and followed by regular administration, as these infants are at increased risk of developing peanut allergy. As there are only data on the preventive introduction of peanuts in infants with mild or no sensitization to peanut in skin prick testing [64], peanut allergy should be excluded in infants with moderate to severe atopic dermatitis before peanuts are introduced. 

### Overweight and obesity 

The recommendation that overweight (body mass index (BMI) percentile > 90 – 97) and obesity (BMI percentile > 97) should also be avoided for reasons of allergy prevention is further supported by recent studies. Prospective cohort studies show that overweight and obesity or a BMI ≥ 30 kg/m^2^ before or at the beginning of pregnancy are positively associated with wheezing [65, 66, 67, 68, 69, 70] and/or asthma [69, 71, 72, 73] in the child. This is confirmed by a pooled analysis of 14 cohort studies including 85,509 children [74] as well as a current meta-analysis [75] that included 22 observational studies with 145,574 mother-child pairs. 

Mediators or influencing factors under discussion for these associations include genetic factors and inflammatory processes as well as the subsequently increased child’s BMI [69, 70, 76]. Rapid weight gain in the first 2 years of life, for example, is associated with an increased risk of wheezing or asthma later in childhood [73, 77, 78, 79]. Cohort [80, 81, 82] and case-control studies [83, 84] confirm results of cross-sectional studies [85, 86, 87, 88, 89, 90] that observed a relationship between overweight/obesity and the occurrence of wheezing or asthma in children. 

While two cross-sectional studies [85, 90] and one cohort study [81] showed a positive association between BMI and asthma only for girls, a meta-analysis [91], however, showed that overweight boys had a higher risk of asthma. 

Some cross-sectional studies also show positive associations of the BMI with eczema [89, 92] and allergic rhinitis [92, 93] in childhood and adolescence. However, prospective birth cohorts do not provide evidence for associations of a high BMI in women before/at the beginning of pregnancy and an increased incidence of other atopic manifestations such as rhinitis and atopic eczema [71, 72]. 

Promoting normal weight development during childhood and adolescence is of great importance not only with regard to allergy prevention, but also for the health of the child in general. Maternal overweight and obesity at the beginning of pregnancy are, among other things, associated with birth complications, gestational diabetes, and later overweight of the child [94]. For all these reasons, women of childbearing age should avoid overweight or get as close as possible to normal weight before pregnancy. 

### Prebiotics and probiotics 

Data from partly large, randomized, double-blind intervention studies consistently show no preventive effects of prebiotics and probiotics for the endpoints allergic rhinitis and bronchial asthma. The vast majority of current intervention studies also show no preventive effect of an administration of supplements with prebiotics and/or probiotics on atopic eczema. Allergy-preventive effects for atopic eczema have been described in a few intervention studies and in cohort studies. However, in some of these studies, allergy-preventive effects were not stable over time. The allergy-preventive effects described in single intervention studies were also not reproducible in the few studies that used the same preparation and study design. In an intervention study describing an allergy-preventive effect for atopic eczema, a significantly higher rate of allergic rhinitis was found as an adverse effect. Published studies using prebiotics and/or probiotics are also characterized by a high degree of heterogeneity regarding the intervention (preparation, dose, duration and time of administration), the studied population, and the time at which individual endpoints were recorded. 

Since 2014, a total of three intervention studies with prebiotics have been identified, all of which used postnatal supplementation. Using a multicenter design, Boyle et al. [95] investigated the use of a partially hydrolyzed whey infant formula enriched with prebiotics (GOS & FOS & pAOS) in a large study group. Compared to cow’s milk formula, the intervention showed no effect on the prevalence of atopic eczema between the ages of 12 and 18 months. Also Ranucci et al. [96] found in an intervention study with 400 infants randomized to prebiotic (GOS/PDX) or standard infant formula after 36 and 48 weeks no effect on the frequency of eczema. In agreement with these data, in a study in Spain including 365 infants, Sierra et al. [97] also found no effect of an infant formula with added prebiotics (GOS) on eczema prevention. The intervention began in the 2^nd^ month of life, and the children were followed up to 12 months of age. The endpoint in this study was a combined endpoint of atopic eczema & wheezing & food allergy. This endpoint was (insignificantly) more often observed in the intervention group (OR 1.56; 95% CI 0.89 – 2.73). In another paper [98] it was shown that the partially hydrolyzed infant formula with added prebiotics (GOS & FOS & pAOS) used in the intervention study by Boyle [95] modulated the intestinal microbiome in such a way that it was more similar to the microbiome of breastfed infants. 

Since 2014, a total of four intervention studies with probiotics have been identified, all of which used supplementation in the pregnant woman and/or mother. Three of the four papers reported clinical endpoints. In a Norwegian follow-up study [100] of the ProPACT study by Dotterud et al. [99], at the age of 6 years there were no significant differences between children of the former placebo (n = 82) and probiotic group (n = 81) with regard to the prevalence of asthma, allergic rhinitis, and atopic dermatitis after administration of *Lactobacillus rhamnosus GG, Bifidobacterium animalis, L. acidophilus* from the 36^th^ week of gestation up to the 3^rd^ month after birth (to the then breastfeeding mother). An unadjusted logistic regression showed a protective effect for atopic dermatitis (OR 0.48, 95%CI 0.25 – 0.92). The number of mothers who needed to be supplemented to prevent atopic dermatitis in one child (number needed to treat) was n = 6. However, this analysis was carried out without adjusting for possible confounders. Another paper by Rø et al. [101] included a subgroup of the ProPACT study (n = 72 (placebo) and n = 68 (actively treated)) to investigate whether probiotic supplementation had an effect on T cells and whether the protective effect on the prevalence of atopic dermatitis observed by Dotterud et al. [99] can possibly be explained by this. The primary aim of this sub-analysis was to investigate in vitro effects on the T-cell population. It was shown that perinatal maternal supplementation with the probiotic combination used (*Lactobacillus rhamnosus GG, Bifidobacterium animalis, L. acidophilus*) led to a reduction in the Th22 cell population compared to placebo. This effect was also observed in children who did not develop atopic dermatitis during the 2-year follow-up period. In the group who developed atopic dermatitis, the proportion of the Th22 cell population was increased. The authors conclude that immunological effects of probiotics may be partially mediated through the reduction of Th22 cells. 

A cohort study from Norway showed a very weak inverse association between the consumption of probiotic milk and milk products (with lactic acid and bifidobacteria) during pregnancy and the risk of eczema in children aged 6 months (OR 0.94; 95% CI 0.89 – 0.99); however, at the age of 18 months this association was no longer observable (OR 1.0; 95% CI 0.95 – 1.05) [102]. 

Numerous other intervention studies that have examined the use of probiotic supplementation in the mother during pregnancy and in the child after birth, or studies that have used supplementation only in the child as an intervention, also show, in most cases, no protective effect on atopic eczema [103, 104, 105, 106, 107]. 

In a large randomised placebo-controlled study from Finland with 1,223 pregnant women, supplementation of a mixture of pre- and pobiotics (LGG,* L. rhamnosus, Bifidobacterium breve Bb99, Propionibacterium freudenreichii ssp. shermanii* JS, and GOS) significantly reduced atopic eczema in children at 2 years of age [108]. In this study, supplementation was started in pregnant women with a probiotic preparation or placebo 2 to 4 weeks before delivery and was continued in the infants with the same probiotics plus GOS or placebo for the first 6 months. At the age of 5 years, no difference was found in the frequencies of eczema. In a post-hoc defined group of children who had been delivered by caesarean section, less IgE-associated allergic disease were observed [109]. In a follow-up of this population at the age of 10 years, in which almost 80% of the original study population participated, it was found that children from the probiotic group had allergic rhinoconjunctivitis significantly more often between the ages of 5 and 10 than the placebo group (36.5% vs 29.0%, OR: 1.43; 95% CI: 1.06 – 1.94; p = 0.03). This difference was seen, however, only in vaginally delivered children (38.6% vs 25.8%, OR: 1.80; 95% CI: 1.29 – 2.51; p <0.01), but not in children after cesarean delivery [110]. In a paper by Hrdý et al. [111], immunological effects after probiotic supplementation (*Escherichia coli* O83: K24: H31) in newborns were described and discussed as a possible explanation for the observed preventive effects on allergic diseases (rhinoconjunctivitis, asthma, atopic eczema). 

In an intervention study by Wickens et al. [112] from New Zealand, *Lactobacillus rhamnosus* HN001 supplementation was compared to supplementation with *Bifidobacterium animalis subsp lactis* HN019 in pregnant women starting from the 35^th^ week of gestation and for another 6 months during breastfeeding as well as in the first 2 years of the child’s life. In this study, a specific effect of HN001 was observed at the age of 6 years on the cumulative prevalence of atopic eczema (HR = 0.56, 95% CI 0.39 – 0.80) and allergic sensitization as shown in the skin prick test (HR = 0.69, 95% CI 0.48 – 0.99) [112]. However, the point prevalence at this point in time showed no significant difference for atopic eczema (RR = 0.66, 95% CI 0.44 – 1.00) and allergic sensitization (RR = 0.72, 95% CI 0.53 – 1.00). For the second probiotic examined in this study, *Bifidobacterium animalis subsp lactis* HN019, no significant effects could be shown [112]. Wickens et al. [113] observed a protective effect of HN001 on the cumulative eczema prevalence even at the age of 11 years. However, when supplementation was restricted to mothers (from the 14^th^ to the 16^th^ week of gestation and 6 months postpartum), there was no preventive effect on eczema, wheezing, or atopic sensitization in children aged 12 months [114]. 

The first intervention study that showed a significant protective effect of probiotics against atopic eczema was published in Finland in 2001 [115]. For this study, a population with an increased allergy risk had been recruited, i.e., women who either themselves or their partners had an atopic disease. The women were supplemented with *Lactobacillus* GG or placebo during pregnancy and the children received *Lactobacillus* GG or placebo during the first 6 months of their lives. After 2 years, there was a significantly reduced prevalence of atopic eczema in the probiotic group (RR 0.51 [95% CI 0.32 – 0.84]). This effect could not be confirmed in a study identical in design that investigated an identical probiotic in a German high-risk population (RR 0.96 [95% CI 0.38 – 2.33]) [116]. In the meantime, a third intervention study with a comparable design has been published, in which the initial data from Finland could not be reproduced either (RR 0.95 [95% CI 0.59 – 1.53]) [107]. However, in this study, only the infants were supplemented with probiotics and not the mothers during pregnancy. 

The discussion about the effectiveness of prebiotics and probiotics is increasingly accompanied by a controversy about the safety and tolerability of individual preparations. It has to be considered among other things, that the studied populations, such as newborns, are a particularly vulnerable group in which the administration of probiotics could lead to infections and sepsis. In addition, attention is drawn to the risk of plasmid-borne antibiotic resistance [117, 118]. In view of the limited effectiveness of the preparations examined, aspects of safety must therefore also be included in the evaluation of prebiotics and probiotics with regard to allergy prevention. 

### Vitamin D supplementation 

The preventive administration of vitamin D in population groups such as breastfeeding women, toddlers, and older children did not indicate a positive effect on the development of allergic diseases in children. 

Also interventional studies including women during pregnancy do not indicate a preventive effect of vitamin D supplementation on the prevalence of atopic eczema, allergic rhinitis, or food allergies in their children [310]. 

The problem with all these studies is the heterogeneity of the populations examined, the number of subjects studied, the period of investigation and the geographical location (impact of UV exposure). Another major issue that complicates the interpretation of the data is the different baseline vitamin D status (measured as serum 25-OH-D values) of the study populations. Two observations are striking: (1) 25-OH-D values below 25 – 30 or below 50 nmol/L at baseline were partly associated with an increased risk of allergic diseases and (2) serum 25-OH-D values > 75 nmol/L (30 ng/mL) were associated with a lower risk. To definitely clarify the question of primary prevention, further studies are required that take into account both vitamin D supplementation during pregnancy and in the postnatal phase without discontinuation. Recommendations cannot be made at the moment. There is contradictory evidence regarding the association between vitamin D supplement intake during pregnancy and the development of allergic diseases in childhood. For example, a combined analysis of two placebo-controlled intervention trials suggests that administration of 4,000 and 2,400 IU of vitamin D per day in pregnancy was only associated with a lower risk of asthma/wheezing in children aged 3 years if the pregnant women had vitamin D levels ≥ 30 ng/mL (75 nmol/L) at baseline [119]. 

In an intervention study by Chawes et al. [120], 623 pregnant women received a placebo-controlled supplementation of 2,400 IU vitamin D/day starting at 24 weeks of gestation until 1 week postpartum, in addition to the standard supplementation of 400 IU/day. During the first 3 years, wheezing was observed in 16% of the children of those mothers who had received the additional vitamin D supplementation, while 20% of the children whose mothers had received a placebo developed wheezing. This difference was, however, not significant. Another randomized, placebo-controlled study from the USA examined 876 pregnant women who received 4,000 IU vitamin D/day after the 10^th^ – 18^th^ weeks of gestation until delivery in addition to the standard dose of 400 IU. Out of the children born to mothers who had received the additional supplementation, 24.3% developed asthma until the age of 3 years, compared to 30.4% in the control group. This difference was also not significant [121]. There were no adverse effects observed in either study. 

Follow-ups to the interventions by Chawes et al. [121] and Litonjua et al. [120] also failed to show an effect of high-dose vitamin D supplementation during pregnancy on the endpoints asthma and wheezing in children aged 6 years [122, 123]. 

An analysis of long-term observations of a birth cohort showed that maternal vitamin D serum concentrations < 50 nmol/L between the 16^th^ and 20^th^ week of gestation were associated with an increased risk of wheezing and asthma (the latter only in boys) at the age of 6 years. The authors conclude that vitamin D supply during pregnancy is important for fetal lung development [124]. In other birth cohorts, it was observed that vitamin D intakes during pregnancy far below the D-A-CH reference values were associated with an increased risk of childhood asthma and allergic rhinitis [124, 125]. Another cohort study that assessed dietary and supplemental vitamin D intake in the 25^th^ week of gestation showed no clear association between vitamin D intake and asthma or allergic rhinitis at 18 months or 7 years of age [126]. 

The overall evidence for effects of vitamin D supplementation in children is also contradictory. For example, a case-control study, conducted as part of a Finnish birth cohort study, showed that children who had a higher vitamin D intake from (fortified) foods and supplements during their first 4 years of life had an increased risk of bronchial asthma by the age of 5 years [127]. However, the differences in vitamin D intakes between cases and controls were very small, so the results should be viewed with caution. In addition, children from the control group had a higher gestational age, had been breastfed longer, and less frequently had parents who were affected by allergies. 

A recent placebo-controlled study in children with asthma was able to show that the supplementation of 4,000 IU vitamin D per day did not affect the time to the next exacerbation [128]. 

### Supplementation of other vitamins 

With the exception of studies on vitamin D supplementation, there are only a few studies examining the impact of vitamin supplementation (vitamins A, E, C, and folic acid) during pregnancy or in infancy on the risk of developing an atopic disease. The available study data do not provide sufficient proof that supplementation of these vitamins is associated with the development of atopic diseases: Two older systematic reviews with meta-analyses [129, 130] came to contradictory conclusions about possible associations between intakes of vitamin C, vitamin A, beta-carotene, and vitamin E or corresponding serum concentrations and the occurrence of asthma or wheezing. A more recent cross-sectional study [131] observed that higher intakes of vitamin C and E (from food and supplements) were associated with a lower prevalence of asthma in children aged 3 – 6 years. In contrast, a prospective cohort study [132] found that the intake of vitamins A and E from supplements (but not from food) during pregnancy was associated with an increased risk of wheezing in the child at the age of 18 months. Also a higher vitamin K intake from food and supplements during pregnancy was positively associated with the prevalence of asthma at the age of 7 years. However, those data do not allow reliable conclusions. 

Given the recommendation to supplement folic acid before and during pregnancy to reduce the risk of neural tube defects, several prospective cohort studies [133, 134, 135] investigated whether there is an association between folic acid supplementation or intake of folate equivalents or the folate status of the expectant mother and wheezing, asthma, or atopic dermatitis in the child. The available study results indicate that folate equivalent intake or folate status during pregnancy are not associated with the development of asthma, wheezing, or dermatitis in children up to the age of 3 years. This conclusion was also drawn in an earlier systematic review [136]. Two later studies [137, 138] indicated that folic acid supplementation, depending on the onset (3^rd^ trimester vs. 1^st^ trimester or earlier) and duration of intake, might increase the risk of developing childhood asthma. However, these data have to be interpreted with caution and were not confirmed by two recent intervention studies [139, 140]. It has also been indicated that the impact of folic acid supplementation on the child’s lung function could be modified by a gene polymorphism of the methylenetetrahydrofolate reductase (MTHFR C677T polymorphism) in the pregnant woman [133]. 

There are no studies relevant for Germany that have examined the impact of vitamin supplements in infants or children on the risk to develop an atopic disease in later life. Intervention studies [141, 142] conducted with infants in Guinea-Bissau (West Africa) indicate that supplementation of high doses (25,000 or 50,000 IU, equivalent to 7.5 or 15 mg) of vitamin A in the first few days after birth may be associated with an increased risk of atopic diseases between the ages of 3 and 9 years, particularly in girls. In another intervention study, vitamin A supplementation of 100,000 IU (= 30 mg) after the 6^th^ month of life had no effect on the child’s risk of atopic diseases [143]. Since healthy infants in Germany usually do not receive vitamin A supplements (in such high doses) and the other conditions in Germany also differ from those in Guinea-Bissau, the relevance of these studies for the derivation of a guideline recommendation for Europe is questionable. Irrespective of this, further studies are necessary in order to be able to better assess the risk of vitamin A supplementation in infancy for the development of atopic diseases in children. 

In summary, based on the available study data, no reliable conclusion can be made about a relationship between the intake of vitamins A, C, E, and K (from food and/or supplements) during pregnancy and the risk of asthma in the child. These vitamins should not be supplemented with the aim of allergy prevention. According to the available studies, periconceptional supplementation of folic acid is not associated with an increased risk of atopic diseases in the child, as long as it is carried out according to the recommendations (4 weeks before pregnancy and in the 1^st^ trimester). 

### Supplementation of omega-3 fatty acids (EPA, DHA) 

Due to the substantial heterogeneity of studies published to date, no conclusive recommendation can be given for supplementation of long-chain, polyunsaturated Ω-3 fatty acids (Ω-3 LCPUFAs) in pregnant women, breastfeeding women, and infants to prevent allergies. Overall, the studies differ significantly in terms of intervention (dosage, content of eicosapentaenoic acid (EPA)/docosahexaenoic acid (DHA), treatment period), the study population examined, and study duration. While some intervention studies, especially those using low-dose supplementation (usually <1 g Ω-3 LCPUFAs), show no effects, other intervention studies that used higher doses (usually ≥ 2.4 g Ω-3 LCPUFAs) show a protective effect of supplementation of Ω-3 LCPUFAs during pregnancy or pregnancy/lactation on the development of allergic diseases in the child, especially with regard to asthma and wheezing. A recent EAACI position paper on a possible impact of the intake of fatty acids on the risk of allergic diseases indicates that an improved Ω-3 LCPUFA supply seems to be decisive for the allergy-preventive effect of supplementation; however, not only the dose but also the bioavailability and the incorporation of the Ω-3 LCPUFAs into the cells, which can vary greatly in the case of supplementation, have to be taken into account [144]. In addition to the heterogeneity of the studies, this makes a conclusive assessment and pooled analyzes more difficult. These aspects were also discussed in two recently published systematic reviews and meta-analyses, which found no significant risk reduction of allergic diseases (atopic eczema, asthma, allergic rhinitis) in childhood with supplementation of Ω-3 LCPUFAs during pregnancy [1, 145, 146] but showed a positive trend in terms of a protective effect on the development of asthma/wheezing [1, 145]. Overall, only few studies assessed the Ω-3 LCPUFAs supply of the mother or the child before the start or during the course of the intervention. However, these studies consistently show that low Ω-3 LCPUFA levels are associated with an increased risk of allergic diseases in the child. This risk could be reduced if the Ω-3 LCPUFA levels were improved by supplementation. This points to a possible individualized or target group-oriented recommendation for supplementation with Ω-3 LCPUFAs in the case of poor supply of DHA and EPA during pregnancy, lactation, or infancy. In order to formulate conclusive and specific recommendations, however, further studies are necessary, which, for example, include explicitely women with a poor supply, or which assess the Ω-3 LCPUFA status in both mother and child at baseline and during the course of the intervention. 

A number of recent observational studies, some with long-term follow-up, also provide evidence for an association between a better supply of Ω-3 LCPUFAs in mothers and their children and a less frequent occurrence of allergic diseases: In a cross-sectional study, Maslova et al. [147], for example, observed an inverse relationship between Ω-3 and Ω-6 LCPUFAs in maternal blood during pregnancy and asthma in middle childhood, and between Ω-3 and Ω-6 LCPUFAs in umbilical cord blood and wheezing in early childhood. A cohort study from Germany showed no significant association between Ω-3 and Ω-6 LCPUFA concentrations or Ω-6/Ω-3 LCPUFA ratios in umbilical cord blood and the frequency of eczema, asthma, hay fever, allergic rhinitis, or sensitization to inhalant allergens in children. However, it was observed that children who had developed eczema by the age of 2 years had significantly lower Ω-3 LCPUFA concentrations and higher Ω-6/Ω-3 ratios. In addition, significantly lower Ω-3 LCPUFA concentrations were measured in 6-year-old children with asthma [148]. Also, in a Swedish cohort, higher plasma concentrations of Ω-3 LCPUFAs in children aged 8 years were inversely associated with the prevalence of asthma and allergic rhinitis at 8 years of age and with a reduced risk of developing asthma between 8 and 16 years of age [149]. 

Since 2014, data from six intervention studies with pregnant and breastfeeding women and one intervention study with infants have been published that investigated the impact of supplementation with Ω-3 LCPUFAs during pregnancy or during pregnancy/breastfeeding on the risk of atopic diseases: In a large Danish study, pregnant women were randomized to a fish oil (2.4 g Ω-3 LCPUFAs/day; 55% EPA, 37% DHA) or placebo group (olive oil) and supplemented accordingly from the 24^th^ week of gestation until 1 week after delivery. Children in the fish oil group showed a significantly lower risk of persistent wheezing or asthma at the age of 3 – 5 years compared to the control group (n = 695). A sub-analysis showed that the preventive effect of supplementation was most evident in children born to mothers whose pre-intervention EPA and DHA blood levels were in the lowest tertile. There were no significant group differences with regard to the occurrence of eczema or allergic rhinitis at the age of 5 years [150]. Another intervention study from Denmark examined the impact of supplementation during pregnancy on allergic respiratory diseases up to young adulthood. In this study, pregnant women in the 3^rd^ trimester were also supplemented with either fish oil (2.7 g Ω-3 LCPUFAs/day; 32% EPA, 23% DHA) or olive oil (n = 396) compared to a capsule without oil (control), and the children were followed up to the age of 24 years. There was a significantly reduced probability of being prescribed asthma medication and receiving a discharge diagnosis of asthma in the fish oil group compared to the olive oil group. With regard to the prescription of medication for allergic rhinitis, there was a lower risk in the fish oil group, but without statistical significance [151]. 

A Swedish intervention study from 2011 examined supplementation during pregnancy and breastfeeding: 145 pregnant women with an increased allergy risk took daily capsules with 2.7 g Ω-3 LCPUFAs (1.6 g EPA, 1.1 g DHA) or soybean oil as a placebo from the 25^th^ week of gestation until the 3^rd^ month of breastfeeding. A protective effect of maternal supplementation on the cumulative incidence of IgE-mediated allergic diseases (eczema, food allergy, asthma, or rhinoconjunctivitis) was observed within the first 2 years of life. In addition, it was found that, regardless of the group, higher DHA and EPA plasma levels in the mother and child were less frequently associated with allergic diseases within the first 2 years of life; this effect was dose dependent [152]. A more recent sub-analysis of this study examined whether the observed preventive effect can also be explained by increased concentrations of Ω-3 LCPUFA in breast milk due to supplementation during pregnancy and lactation. It was shown that the supplementation led to significantly higher concentrations of Ω-3 LCPUFAs in breast milk and to a preventive effect on the occurrence of IgE-mediated diseases in the child [153]. 

In contrast, three other intervention studies showed no allergy-preventive effects of supplementation with Ω-3 LCPUFA during pregnancy. However, in comparison to the studies described above, in some cases lower amounts were supplemented and serum concentrations were not measured before or during the intervention: For example, in a large Australian intervention study no differences were found in children at risk of atopic disease (n = 668) with regard to the development of IgE-mediated allergic diseases (atopic eczema, IgE-mediated wheezing, allergic rhinitis, or rhinoconjunctivitis) at the age of 6 years, following maternal supplementation of 900 mg fish oil (800 mg DHA, 100 mg EPA) or placebo (vegetable oil) daily from the 21^st^ week of gestation until birth [154]. A randomized study from Mexico also found no protective effect of supplementation with 400 mg algae-based DHA compared to placebo (cereal and soybean oil) in pregnancy on the occurrence of wheezing up to the 18^th^ month of life (n = 869) [155]. In another study from the USA with a comparatively small number of cases (n = 84), which primarily examined the effect of supplementation with Ω-3 LCPUFAs (fish oil) on the prevention of depression during pregnancy, both an EPA-rich (1,060 mg EPA and 274 mg DHA/day) and a DHA-rich (900 mg DHA and 180 mg EPA/day) prenatal supplementation were associated with an increased risk of developing atopic eczema at the age of 36 months [156]. 

In line with the data available for pregnancy and lactation, an Australian study from 2012 had already shown that a daily dose of 650 mg fish oil (280 mg DHA, 110 mg EPA) compared to placebo (olive oil) administered to infants at an increased risk of allergies in the first 6 months of life resulted in significantly higher Ω-3 LCPUFA concentrations at the age of 6 months. Although postnatal supplementation was associated with a potentially positive effect on infant immune function at 6 months of age [157], there was no difference in the prevalence of eczema or other atopic diseases between the intervention and control groups. However, regardless of the group, high levels of Ω-3 LCPUFAs in the child’s blood were associated with a lower risk of eczema and wheezing [157, 158]. 

Since 2014, two further studies have been identified that examined whether feeding infants with Ω-3-LCPUFA-fortified infant formula (but without measuring the LCPUFA serum level in the child) may reduce the risk of atopic diseases in later childhood: In an Australian intervention study, 657 preterm infants were fed an infant formula highly fortified with DHA (DHA: 1% of the fatty acids) compared to an infant formula with standard DHA content (DHA: 0.3% of the fatty acids), while at the same time breastfeeding mothers received 0.5 g fish oil (900 mg DHA and 195 mg EPA) (or soybean oil as a placebo) to increase the DHA concentration in the breast milk. At the age of 7 years, there was no difference between the groups with regard to the development of asthma/wheezing, hay fever, and eczema [159]. A French prospective observational study including 325 newborns who were either fed an infant formula fortified with DHA and arachidonic acid (ARA) (17 mg DHA/100 kcal and 34 mg ARA/100 kcal) or an infant formula without these fatty acids also showed no significant differences regarding the occurrence of eczema in the 1^st^ year of life [160]. 

No side effects were reported in the intervention studies described here on supplementation of Ω-3 LCPUFAs for allergy prevention. According to EFSA, there are no safety concerns regarding the daily intake of Ω-3 LCPUFAs via supplements for adults up to a combined dose of 5 g EPA and DHA or up to a dose of 1.8 g EPA alone or up to a dose of 1 g DHA alone for healthy adults [161]. Regardless of the purpose of allergy prevention, German professional societies recommend DHA supplementation during pregnancy and breastfeeding in order to achieve the recommended average daily intake of at least 200 mg DHA if no oily sea fish is consumed [94, 162]. According to the EFSA, the appropriate daily intake for infants up to the age of 6 months is 100 mg DHA [163]. According to the Delegated Regulation (EU) 2016/127, a mandatory addition of DHA of at least 20 mg/100 kcal and at most 50 mg/100 kcal is stipulated for infant formula and follow-on formula. 

### Pets 

Various epidemiological studies showed that having a dog in the household during the first years or the first 3 years of a child’s life has a primary protective effect with regard to the development of food allergies [164], airborne allergies, and bronchial asthma at school age (6 – 13 years) [165, 166, 167, 168, 169]. A Swedish birth cohort study [169] found no protective association of dogs in the household during the 1^st^ year of life and allergic rhinitis at the age of 13 years. With regard to keeping cats or other typical pets, there are still conflicting data. In the Swedish BAS cohort, keeping cats in the 1^st^ year of life turned out to be protective with regard to the development of allergic rhinitis at 13 years of age, but these data are based on parent questionnaire and not on a physician’s diagnosis [169]. In a large Dutch study, no association was found between pet exposure in childhood and bronchial asthma at 17 years of age [170]. It remains to be seen whether keeping a dog should be explicitly recommended for families as a primary allergy prevention. Controlled studies are lacking. However, the evidence of protection through early contact with dogs has become very clear in the last 6 years. An influence on the child’s microbiome can be assumed. 

### House dust mites, endotoxins in house dust 

Earlier studies could not demonstrate reliable effectiveness of the reduction of the domestic allergen content as a measure of primary allergy prevention. Studies on the correlation of early exposure to house dust mites, animal dander, and endotoxins and the later development of asthma and/or allergic sensitization show partially contradictory results [171, 172]. In the U.S. birth cohort study URECA, exposure to house dust mite allergens in the 1^st^ year of life was not associated with an increased risk of recurrent wheezing at 3 years of age [173]. The microbial content of the house dust was also examined in a sub-sample [173]. A combined analysis of this nested case-control study showed the lowest rates of allergic sensitization and wheezing at age 3 years in the group of children exposed to high levels of both indoor allergens and bacterial endotoxins during the 1^st^ year of life, indicating a synergistic effect. In a Finnish birth cohort study, a sub-analysis assessed the associations between quantity and diversity of microbial markers in house dust samples collected at 2 months of age and later respiratory symptoms and allergies in children [174, 175]. The microbial diversity correlated negatively with the asthma risk at 6 years [174] and at 10.5 years [175]. Other studies found differences in the qualitative and quantitative composition as well as the microbial diversity of house dust from homes in which children grow up with and without asthma symptoms [176, 177, 178]; in particular, the indoor microbiota in poorer urban residential areas (lower richness, lower Shannon index, specific germ clusters) appear to be associated with a risk for the development of respiratory symptoms. Further research is necessary to clarify whether the child’s microbiome is altered as a result and to better characterize protective factors and risk factors. 

### Allergen-specific immunotherapy (AIT) 

Allergen-specific immunotherapy (AIT) is a form of therapy that has been well documented in terms of its effectiveness in allergic rhinitis and/or allergic bronchial asthma. The indication is to be made according to the AWMF guideline. 

The disease-modifying effect of AIT comprises:

- Prevention of new IgE sensitizations [179, 180]
- Prevention of bronchial asthma by AIT in allergic rhinitis and allergic rhinokonjunctivitis [179, 180]

Some studies have investigated the prevention of new IgE sensitizations by AIT, mostly in children with allergic rhinitis/ allergic rhinokonjunctivitis or asthma [181, 182, 183, 184, 185, 186, 187, 188, 189]. Two studies were placebo-controlled and were carried out in atopic infants [190, 191, 192] or in IgE-sensitized, non-allergic children [193]. It has been indicated that primary preventive AIT with house dust mite extract can prevent new sensitizations in the first 2 years of life. A modifying effect on allergic symptoms has not been reported [179]. 

The prevention of bronchial asthma by AIT in allergic rhinitis/allergic rhinokonjunctivitis was considered to be secondary/tertiary prevention at best and is not discussed in detail in the present guideline. A small double-blind, placebo-controlled study [194] in patients with perennial rhinitis due to house dust mite allergy showed not only improvement in bronchial hyperreactivity but also a significantly lower rate of asthma after 2 years of AIT compared to the control group (0 vs. 9%). The significantly larger PAT study [188] also showed a clear effect in terms of a reduced risk of developing asthma after 3 and 10 years of follow-up in children with seasonal allergic rhinitis. Data from a 3-year double-blind, placebo-controlled SLIT study on the treatment of children with allergic rhinitis due to grass pollen failed to meet the primary endpoint (time to first diagnosis of asthma) but showed reduced asthma symptoms and asthma medication use compared to the placebo group after 3 and 5 years. 

### Farms 

On the subject of farms, an updated statement was adopted. This takes the evidence into account that it has repeatedly been shown that exposure to a farm in the first years of life is associated with a reduced prevalence of asthma and also allergic diseases. 

It has been suggested that the reasons for this are (1) a more efficient stimulation of the innate immune system in children exposed to farms compared to those who are not s. (2) a microbial composition of the house dust on farms (with only small amounts of Streptococcaceae) being protective against the development of asthma. Children not exposed to farms are also protected when the microbial composition of house dust is similar to that of the farm environment [195]. Mechanistically, a reduced pro-inflammatory cytokine response can also be seen. 

Exposure to farm milk and to barns shows a protective effect against childhood asthma. The better function and higher number of regulatory T cells [196], which are functionally active up to the age of 4.5 years [197] are regarded as the cause here. On the other hand, increased amounts of omega-3 polyunsaturated fatty acids [198] and increased exposure to non-microbial N-glycolyneuraminic acid (Neu5Gc) have been discussed as playing a role in the protective farm effect [199]. 

Not only in Europe but also in South Africa, exposure to animals has been shown to be strongly protective against allergic diseases in rural areas, while in the cities the consumption of fermented milk in particular is protective [200]. 

### Caesarean section 

Already in the last version of the guideline it was stated that the evidence of the studies available at that time showed an increased risk, for asthma, in children who were born through an elective caesarean section. In the meantime, further studies have been published that reinforce the recommendation made at that time with a higher level of evidence and also a higher level of recommendation. It has also been shown that children, especially after elective caesarean sections, have an increased risk of asthma in infancy and at school age. 

A meta-analysis of 9 European cohort studies showed a significantly increased risk of developing asthma in patients aged 5 – 9 years (adjusted OR 1.49; 95% CI 1.13 – 1.97). Other international studies with different designs came to the same conclusions [201, 202]. In particular, a large population-based study with more than 136,098 children from the USA [203] also confirmed this result (adjusted OR 1.11; 95% CI 1.06 – 1.15). 

A German prospective birth cohort study could not show any significant results at the age of 15 years (OR: 0.87 (95% CI 0.57 – 1.33). The same applies to a Brazilian cross-sectional study [204]. In both studies, however, no distinction was made between elective and secondary or emergency caesarean section. 

Most studies assume that the protective effects of natural birth are due to a change in the infant microbiome. Particularly in elective caesarean section there is no transmission of the maternal microbiome to the child. 

The current literature does not suggest that a reduction of other allergic diseases is possible by avoiding a caesarean section. Studies on atopic dermatitis and on rhinitis allergica in particular did not show any correlation with the mode of birth [205]. 

### Vaccinations 

Data from various cohort studies show neither increases nor reductions of allergic sensitizations, atopic eczema, or bronchial asthma after vaccinations in infancy [206, 207]. 

A better vaccination coverage rate (higher number of vaccine doses received) is associated with a lower probability of allergic sensitizations [208], allergic rhinoconjunctivitis [209], and/or bronchial asthma [208]. Lower prevalences [208] and lower severities [206] were observed in atopic dermatitis. 

A delayed primary immunization also showed inconsistent results in several cohort studies with regard to the development of allergic diseases [210, 211]. 

Changing the pertussis vaccine from a cell-based to an acellular vaccine was not associated with an increased risk of food allergies or allergic diseases [213, 214, 215]. 

In a randomized trial, BCG vaccination within 7 days after birth was associated with a lower risk of atopic eczema in children with an atopic disposition [216]. On the other hand, there was no effect on recurrent wheezing in the first year of life [217], on allergic sensitization, and on suspected food allergy up to the 13th month of life [218] or on the number of hospitalizations [219]. 

Various retrospective cohort studies showed no effect of previous routine BCG vaccinations on the development of sensitizations, hay fever, asthma, or eczema in childhood [220, 221]. 

In utero exposure to influenza vaccination is not associated with bronchial asthma in children [222]. 

### Day care centers 

There is conflicting data on the association between care at day care centers and the prevalence of childhood asthma at different ages so that a recommendation cannot be derived. In this context, the studies did not take into account essential trigger factors for the the later course of asthma, namely infections and allergic allergic sensitisations. 

While kindergarten care in infancy may temporarily lead to an increased prevalence of obstructive respiratory symptoms, presumably due to a higher number of viral infections [223], retrospective data show a protective effect on bronchial asthma in school children [223], but also increases of prevalences [224]. Therefore, no recommendation can be derived from the small number of studies with retrospective data collection so far, even if the number of cases is high. Further studies are necessary addressing the presumably relevant influencing factors – very early admission to care before the 6th/12th months of life (possibly increasing prevalence), duration of weekly care [224], and development of an allergy to environmental allergens. 

Overall, the effects are not pronounced. For children who started day care after the 12th/18th months of life, the effects were very small or not detectable in the studies mentioned above. 

The data from a study showing an increased prevalence of atopic dermatitis [223] in day-care children are based solely on retrospective statements from parents on that diagnosis during a visit to the doctor. 

With regard to the prevalence of food allergies, no differences were found between children in child care and those who are not [223]. 

### Drugs 

Numerous controlled studies show low associations (mean increases in the RR in the range of 1.2 – 2.5) between drug intake (antibiotics, NSAIDs) and atopic diseases [225, 226, 227, 228, 229, 230, 231, 232, 233, 234, 235, 236, 237, 238, 239, 240]. A distinction has to be made between prenatal intake by the mother and postnatal exposure of the child. The data on the increase in the relative risk of atopic diseases are more extensive for the use of antibiotics [225, 226, 227, 228, 229, 230, 231, 232, 233] than for the use of NSAID [234, 235, 236, 237, 238, 239, 240]. 

In summary, the data show that children whose mothers took antibiotics during pregnancy have an increased mean risk of developing asthma (11 studies, RR 1.3 – 3.2) [225, 226, 227, 228, 229, 230, 231, 232, 233, 235]. Studies (mainly birth cohorts) on the exposure of children in their 1st or 2nd years of life show a slightly increased risk of allergic asthma in the case of postpartum exposure to antibiotics, and a less increased risk of allergic rhinitis or of eczema later in life. A study from Germany showed an age-dependent effect with regard to atopic dermatitis only (birth cohort Pasture, up to the age of 6 years RR AD 2.65) [231]. 

Contradictory data are available intake of paracetamol in the first years of life (birth cohorts and case-control studies) [232, 233, 234, 235, 236] or during pregnancy [237, 238, 239, 240] so that no clear connection can be establishedbetween the intake of paracetamol and atopic diseases. 

Due to potential confounding factors, the available studies on medication intake and atopic diseases should generally be interpreted with caution. 

### Skin barrier 

A disturbed skin barrier often precedes the development of atopic dermatitis and sensitizations to allergens including protein allergens. 6 years ago, two independent, smaller studies (n = 118 and n = 135) described that a consequent application of emollients significantly reduced the incidence of eczema (i.e., between 30 and 50%) in infants from atopic risk families [241, 242]. The main criticism of the studies, which attracted a lot of attention, was the relatively small number of participants. The results could not be reproduced in a larger Japanese study including 800 children [243]. Two negative follow-up studies with even higher case numbers are currently available:: In the BEEP (Barrier Enhancement for Eczema Prevention) study from the UK, 1,394 newborns at high risk of eczema (i.e., at least one first-degree relative with medically diagnosed eczema, allergic rhinitis, or asthma) were recruited, randomized 1:1, and either treated daily with an emollient plus standard skin care regimen (“emollient group”) or with standard skin care regimen only (“control group”) during their first year of life [244]. The primary objective of the study was to assess the frequency of eczema at 2 years of age (defined according to the UK Working Party criteria of AD). By the age of 2 years, eczema had manifested in 139 (23%) of 598 infants in the emollient group and 150 (25%) of 612 infants in the control group. The mean number of skin infections per child was 0.23/year in the emollient group versus 0.15 in the control group [244]. 

In a similar Norwegian study, 2,397 infants were randomized into four groups at birth [245]: (1) controls without advice on specific skin care and with recommendations to follow national infant feeding guidelines (no intervention); (2) skin intervention group (emollients including regular use of bath additives and face cream); (3) food intervention group: early supplemental feeding of peanut, cow’s milk, wheat, and egg; or (4) combined skin and food interventions. The primary outcome to be compared was the onset of atopic dermatitis at 12 months of age. Clinical examinations were performed at 3, 6, and 12 months of age by blinded investigators. Atopic dermatitis was observed in 8% in the no-intervention group, in 11% in the skin intervention group, in 9% in the food intervention group, and in 5% in the combined intervention group, with no significant differences. Thus, neither the use of emollients nor early introduction of complementary foods reduced the development of atopic dermatitis. 

There is still a lack of controlled studies investigating the daily use of emollients in infants and toddlers with visibly dry skin who already have eczema or who have genodermatosis with skin barrier disorders associated with IgE-mediated sensitization (especially ichthyosis vulgaris, Netherton syndrome) – also with the aim of preventing further eczema and allergies. 

### Pollutants 

There is very heterogeneous and weak data on molds and moisture as indoor pollutants. The existing studies are cross-sectional analyzes that were carried out on the basis of information provided by parents. The study by Milanzi et al. [170] (n = 1,871) shows no association between mold/moisture at different time points in childhood and the development of asthma. In contrast, another paper (n = 4,089) shows that exposure to mold and moisture directly correlates with the risk of developing asthma. No impact on rhinitis was found [246]. A small Finnish study (n = 214) observed an increased risk of developing rhinitis and asthma in children [247]. Zhang et al. [248] (n = 36,541) evaluated parent questionnaires and found an increased risk of rhinitis when moisture and molds were present. 

The connection between exposure to tobacco smoke and the development of bronchial asthma has been clearly documented. The evaluated studies show a stringent association between active and passive exposure to tobacco smoke and the development of allergies and, especially, asthma. This mainly affects children from high-risk families [249, 250, 251]. 

Active smoking appears to have little, if any, impact on the development of other allergic diseases such as atopic eczema, or food allergies. There are no high-quality studies on this topic. 

For other *indoor pollutants,* the following papers were found: In a cohort study including 560 children, O’Connor et al. [172] found no association of nitrogen dioxide, ergosterol, and endotoxin in the room air during the first 3 years of life and asthma at the age of 7 years in children from high-risk families. 

On the basis of Danish cohorts, Callesen et al. [171] investigated phthalates, nicotine, and CO_2_ in the indoor air in a sample of atopic children (74 of them asthmatics, 83 with rhinoconjunctivitis, and 90 with atopic dermatitis). An interesting finding was the increased nicotine content in house dust in asthmatic children. With regard to phthalates, no relevant association was seen, except for a higher proportion of DEHP in the homes of children whose parents reported “current wheeze”. 

In a small case control study, Madureira et al. [252] showed an association between the presence of air conditioning, water damage, and visible mold in the previous year in 38 asthmatic children. A large number of analyzed indoor pollutants, such as volatile organic compounds (VOC), PM2.5/10, bacteria, and fungi, did not show any such association apart from D-limonene; however, the range of fluctuation in the measurements was small. 

Two more extensive cross-sectional studies from Portugal [253] and China [254] used surrogates to show a moderate influence of air pollutants on asthma. The Portuguese study showed an association of indoor CO_2_ concentrations (day care centers) with asthma (OR 1.1) and wheezing in the previous 12 months; the Chinese study demonstrated risks of a similar magnitude for atopic respiratory diseases when, e.g., pungent odor and tobacco smell were present. 

Studies proving or excluding an association between exposure to *plasticizers *(such as phthalates) and the development of allergic diseases could not be identified. 

There are (two) conflicting studies on whether regular exposure to *chlorinated pool water* during swimming pool visits could increase the risk of asthma. Both studies do not show a connection between regular swimming pool visits and an increased risk of rhinitis and eczema. 

A cross-sectional study shows an increased risk of asthma, especially in children with atopic sensitization, but no increased risk of rhinitis and eczema due to regular swimming [255]. 

In another study, there was no significant association between early, late, or current swimming/swimming pool visits and asthma, dermatitis, wheezing, allergic rhinitis, and eczema in children up to the age of 12 years [256]. 

### Motor vehicle emissions 

With regard to the influence of indoor and outdoor air pollutants, including exposure to tobacco smoke, the previous recommendations are further supported by the results of current studies. With regard to the exposure to motor vehicle pollutants, data from the different studies show heterogeneous results. In some studies, exposure to nitrogen oxides and particulate matter with a particle size < 2.5 µm during pregnancy was associated with the development of rhinitis and hay fever [257, 258, 259] or asthma [260]. Additionally, data from cohort studies show that increased exposure to traffic-related pollutants was associated with an increased incidence of wheezing [261] or asthma [262, 263]. One study showed that living near a busy road was associated with an increased risk of developing asthma [264, 265, 266, 267]. On the other hand, there are also various studies that do not show an association between exposure to vehicle pollutants and allergic or respiratory diseases [268, 269, 270, 271]. A long-term study from South Korea [272, 273] shows that increased ozone levels also increase the relative risk of acute asthma attacks. 

### Psychosocial factors 

Depression and other types of psychological stress and illnesses in the mother during pregnancy and after birth have been shown in epidemiological meta-analyses [274, 275, 276] to be factors that contribute to the development of an atopic disease. There has been only little research on the influence of the father’s psychological stress. Relationships could be shown for:

- Prenatal maternal stress in the form of negative life events, anxiety/depression, experiencing severe loss and violence, experiencing stress, socioeconomic stress, and work-related stress. These factors showed a positive interaction with allergy-related parameters such as asthma, atopic dermatitis, allergic rhinitis, and IgE [274, 275] in 21 of 25 studies and in 25 of 30 studies, respectively; however, there were strong quality differences in the study design and in the choice of outcome measures between the studies. The results are consistent with an older meta-analysis including 22 studies [276] and indicate that anxiety and depression in the last trimester in particular are associated with negative effects [275].
- Prenatal maternal stress is related to symptoms of atopic dermatitis in the 2-year-old child [277], wheezing in the 1- to 4-year-old child [278, 279], and atopic dermatitis in the 7-year-old child [280].
- Prenatal stressful life events of the mother are related to asthma, allergic rhinitis, and eczema in the 14-year-old child but not in the 6-year-old child if the stress was experienced between the 18^th^ and 34^th^ week of pregnancy [281], as well as in 6-year-old girls [282].
- Prenatal maternal anxiety and depression are related to the development of atopic dermatitis in the 1-year-old child [283], to wheezing episodes in the 1- to 4-year-old child [284], to allergic rhinoconjunctivitis within the first 5 years of life [285], to atopic dermatitis in the 5-year-old child [284], to asthma in the 7-year-old child [286], and to sensitization to inhaled allergens in the 10-year-old child [287].
- Postpartum maternal depression is related to atopic dermatitis in the 12-month-old child [288], in the 3-year-old child [289], to respiratory symptoms in preschool children, with girls being affected 3 times more often than boys [290], and to asthma in the 6- to 8-year-old child [291].
- Maternal lack of sensitivity, anxiety, and apathy is related to atopic dermatitis in the 18-month-old child [286].
- Maternal depression is associated with bronchial asthma and atopic dermatitis in the child [292].
- Use of psychological therapy offers by the mother is related to asthma in the 12-year-old child [293].
- Paternal depression symptoms at any point in life are related to an increased risk of asthma in the 7-year-old child [294].

Epidemiological studies also show a connection between emotional abuse in early childhood as well as traumatic experiences at any point in life and the development of allergy symptoms later in life [295]. In addition, an interaction between depressive symptoms and other risk factors for allergy, such as obesity, with the severity of allergy symptoms has been shown [296]. Furthermore, a connection between a lack of maternal sensitivity and the development of atopic dermatitis could be shown [286]. At the same time, a protective effect of high maternal sensitivity with regard to the development of atopic dermatitis in children up to the age of 18 months was observed, as was an equally positive effect of social support [286]. 

Complementary to this, numerous epidemiological and prospective studies have shown that the development of allergic diseases might subsequently increase the probability of developing psychological stress and associated diseases [297, 298, 299, 300, 301, 30^2^] (phobias, depression, ADHD), which can possibly be reduced by good disease control but which, on the other hand, can also impair disease control [303]. In the case of comorbidity of allergy and mental illness, a higher disease severity and lower quality of life and the development of further complications can be expected, which can be prevented by simultaneous diagnosis and treatment of mental comorbidity in allergy [304, 305, 306]. 

In animal experiments, stress transmitter-reducing and anti-depressive therapies show good effectiveness in reducing stress, anxiety, and depression in allergy patients [307]. Clinical evidence currently exists for educational programs and anti-depressive drug therapy as well as psychotherapy [28, 51, 308, 309]. 

## Funding 

None. 

## Conflict of interest 

Potential conflicts of interest (COI) were systematically recorded according to the AWMF standards and evaluated by an independent person. Information on the following points was collected: activity as a consultant and/or expert; participation in a scientific advisory board; paid lecturing or training activity; paid authorship or co-authorship; research projects/conduct of clinical studies; owner interests (patent, copyright law, share ownership); indirect interests such as participation in professional societies. The evaluation was carried out according to “Guideline topics affected by COI”, a classification with regard to relevance and the formulation of consequences. The COI of the authors and their rating can be viewed on the AWMF homepage. 

**1. Nutrition. Table1:** 1.1. Maternal nutrition during pregnancy and lactation.

**Level of recommendation**	**Statement**
A	**Statement:** During pregnancy and lactation, a balanced, diverse diet covering all nutritional requirements is recommended. This also includes the consumption of vegetables, milk (products) (including fermented milk products such as yogurt), fruits, nuts, eggs, and fish.
	**Recommendation:** Dietary restrictions (avoidance of potent food allergens) during pregnancy or lactation should not be made for the purpose of allergy prevention (A).
Level of evidence	Studies on the general statement: Celik 2019 (2–); Moonesinghe 2016 (2+); Ogawa 2018 (2+); Oien 2019 (2++); Stratakis 2017 (1++); Rucci 2016 (2++); Leermakers 2013 (2+); Miyake 2013 (2+); Pele 2013 (2+): Gardner 2020 (2+); Miyake 2014 (2+); Chisaguano 2014 (2+); Bunyavanich 2014 (2+), Bedard 2020 (2+), Rosa 2020 (2+)
	Recent studies on the recommendation to avoid dietary restriction are lacking; recommendation is based on previous recommendations and, with regard to food allergy, on the EAACI guideline
Level of consensus	Strong consensus

**1. Nutrition. Table2:** 1.2. Breastfeeding.

**Level of recommendation**	**Statement**
A/B	**Statement:** Breastfeeding has many benefits for both mother and child.
	**Recommendation:** Infants should be breastfed exclusively for the first 4 – 6 months, if possible. (A) Breastfeeding should also be continued when complementary foods are introduced. (A)
	**Recommendation:** Additional feeding of cow’s milk-based infant formula in the first days of life should be avoided if the mother wishes to breastfeed. (B)
Level of evidence	Filipiak-Pittroff 2018 (1+); Quigley 2018 (2+); Den Dekker 2016 (2+), Azad 2017 (2++); Klopp 2017 (2++); Elbert 2017 (2+); Van Meel 2017 (2++), Groenwold 2014 (2++); Ajetunmobi 2015 (2–); Jelding-Dannemand 2015 (2+); Leung 2016 (2–), Nwaru 2013 (2++), Rosas-Salazar 2015 (2–)
	Evidence of EAACI guideline for avoidance of temporary feeding of cow’s milk-based infant formula: Urashima 2019
Level of consensus:	Consensus

**1. Nutrition. Table3:** 1.3. Breast milk substitute and cow’s milk substitutes in children at risk.

**Level of recommendation**	**Statement**
A/B	**Recommendation:** If breastfeeding is not or not sufficiently possible, an infant formula should be given. For children at risk of allergies, it should be checked whether an infant formula with proven effectiveness, demonstrated in allergy prevention studies, is available until complementary food is introduced. (B)
	**Recommendation:** Infant formula based on soy protein is not suitable for allergy prevention and should therefore not be given for this purpose. (A)
	**Comment:** Soy products can be used as part of complementary foods, independently from the purpose of allergy prevention.
	**Recommendation:** Since there is no proof of an allergy-preventive effect of other animal milk, such as goat milk (not even if they are the basis of infant formula), sheep or mare milk, these should also not be given for the purpose of allergy prevention. (B)
	**Comment:** Cereal drinks are no milk substitute from a nutritional point of view.
	**Recommendation:** Since there is no proof of an allergy-preventive effect of cereal drinks, these should also not be given for the purpose of allergy prevention. (B)
Level of evidence	Hypoallergen (HA) hydrolyzed formula: Von Berg 2016 (1++), Davisse-Paturet 2019 (2++)
	Soy formula: no current evidence found
	Milks of other animals: no current evidence found
Level of consensus:	Consensus

**1. Nutrition. Table4:** 1.4. Complementary food and transition to family nutrition.

**Level of recommendation**	**Statement**
A/B/C	**Statement:** There is some evidence that the diversity of the child’s diet in the first year of life has a protective effect on the development of atopic diseases. A divers diet also includes fish and a limited amount (up to 200 mL per day) of milk or natural yogurt and hen’s egg as part of the complementary food.
	**Recommendations:** Depending on the readiness of the infant, the feeding of complementary food should start from the beginning of the 5^th^, at the earliest, to the beginning of the 7^th^ month of life, at the latest. (B)
	There is no proof of a preventive effect of dietary restriction by avoiding potent food allergens in the first year of life. Therefore, no restriction should be made. (A)
	For prevention of hen’s egg allergy, well-cooked (e.g., baked or hard-boiled), but no “raw” eggs (not even scrambled eggs), should be introduced with the complementary food and given regularly. (B)
	For prevention of peanut allergy, introduction and regular consumption of peanuts in an age-appropriate form (e.g. peanut butter) may be considered in infants with atopic dermatitis living in families with regular peanut consumption. (C)
	A peanut allergy should first be ruled out, especially in infants with moderate to severe atopic dermatitis. (A)
Level of evidence	Studies for the statements on complementary foods regarding diversity, fish, milk (yogurt):
	Crane 2018 (2+); Turati 2016 (2++); Nwaru 2014 (2++); Roduit 2014 (2++); Roduit 2018 (2++); Oien 2019 (2++); Klingberg 2019 (2++); Vasileiadou 2018 (2+); Lumia 2015 (2+); Shoda (2+)
	Recent studies on recommendation to avoid dietary restriction are lacking; recommendation is based on previous recommendations and, with regard to food allergy, on the EAACI guideline
Level of consensus:	Strong consensus

**1. Nutrition. Table5:** 1.5. Body weight.

**Level of recommendation**	**Statement **
A	**Statement:** 1) An increased body mass index (BMI) of the mother before or at the beginning of pregnancy is positively associated with wheezing or asthma in the child, and 2) overweight and obese children are more frequently affected by asthma than normal-weight children.
	**Recommendation:** Overweight/obesity in women before and during pregnancy as well as in children and adolescents should be avoided for asthma prevention. (A)
Level of evidence	Women before and at the beginning of pregnancy: Liu 2020 (1++); Ekstöm 2015 (2+); Eising 2013 (2+); Guerra 2013 (2+); Harpsøe 2013 (2+); Leermarkers 2013 (2+); Harskamp-van Ginkel 2015 (2+); Wright 2013 (2+); Ziyab 2014 (2+); Pike 2013 (Pike 2+); Zugna 2015 (1++)
	Children: Loid 2015 (2++); Ziyap 2014 (2+); Popovic 2016 (2+); Casas 2016 (2++); Tsai 2018 (2+); Ekström 2017 (2+); Nahhas 2014 (2+); Forno 2014 (2-); Lang 2018 (2-)
Level of consensus:	Consensus

**2. Food supplements. Table6:** 2.1. Supplementation of prebiotics and probiotics.

**Level of recommendation**	**Statement**
A	**Background:** Data from partly large-scale, randomized, double-blind intervention studies consistently show no preventive effects of prebiotics and probiotics for the endpoints allergic rhinitis and bronchial asthma. The vast majority of current intervention studies also show no preventive effect of supplementation with prebiotics and/or probiotics on atopic eczema.
	**Recommendation:** Prebiotics and/or probiotics should not be given to pregnant women or infants for the purpose of allergy prevention, not even as part of infant formula. (A)
Level of evidence	Boyle 2016 (1++); Abrahamsson 2013 (1+); Allen 2014 (1++); Loo 2014 (1+); Bertelsen 2014 (2++); Peldan 2017 (1++); Wickens 2013 and 2018a (1++); Wickens 2018b (1+); Ranucci 2018 (1++); Sierra 2015 (1++); Wopereis 2018 (1+); Lundelin 2017 (1+); Cabana 2017 (1-); Niinivirta 2014 (2+); Simpson 2015 (1-); Ro 2017 (1++); Hrdy 2018 (2+); Rutten 2015 (1+); Kim 2015 (1+); Murphy 2019 (1+)
Level of consensus:	Consensus

**2. Food supplements. Table7:** 2.2. Supplementation of vitamin D.

**Level of recommendation**	**Statement**
A	**Background:** Current studies show no protective effect of vitamin D supplementation during pregnancy, breastfeeding or in children with regard to allergy prevention in children.
	**Recommendation:** Pregnant women and healthy infants or older children should not take vitamin D supplements for the purpose of allergy prevention. (A)
	**Statement:** The recommendation established in Germany to supplement infants with vitamin D (400 – 500 IU/day) up to the child’s second early summer remains unchanged.
Level of evidence	Supplementation during pregnancy:
	Wolsk 2017a und b (1+); Zosky 2014 (2–); Maslova 2013 (2–); Litonjua 2016/2020 (1+); Chaves 2016 (2+); Brustard 2019 (2+)
	Supplementation in infancy and early childhood: Nwaro 2017 (2+), Forno 2020 (1+)
Level of consensus:	Strong consensus

**2. Food supplements. Table8:** 2.3. Supplementation of other vitamins.

**Level of recommendation**	**Statement**
A	**Background:** There is insufficient proof that vitamin supplementation (such as A, C, E, K, or folic acid) during pregnancy is associated with prevention or an increased risk of atopic diseases in the child. There are no (reliable) study data on possible effects of vitamin supplementation in infancy.
	**Recommendation:** Pregnant women should not avoid taking folic acid for the purpose of allergy prevention. (A) Pregnant women and healthy infants or older children should not take vitamin supplements for allergy prevention. (A)
	**Statement:** Periconceptional folic acid supplementation according to the recommendations should be performed.
Level of evidence	Supplementation during pregnancy: Maslova 2014 (2++); Roy 2018 (2+); Trivedi 2018; den Dekker 2018 (2+); Crider 2013 (unrated)
	Supplementation in infancy: Aage 2015 (1++); Kiraly 2013a (1+); Kiraly 2013b (1+) [all without relevance for Germany]
Level of consensus:	Strong consensus

**2. Food supplements. Table9:** 2.4. Supplementation of long-chain omega-3 fatty acids (EPA, DHA).

**Level of recommendation**	**Statement**
	**Background:** Due to the heterogeneity of the study data, no final recommendation can be given for supplementation of Ω-3 LCPUFAs for allergy prevention in pregnant women, breastfeeding women and infants.
	**Statement:** Some studies show that low serum levels of Ω-3 LCPUFAs in pregnant women, breastfeeding women, and infants were associated with a higher risk of allergic diseases in the child, especially of asthma and wheezing, and that this risk may be reduced by supplementing Ω-3 LCPUFAs.
Level of evidence	Bisgaard 2016 (1++); Warstedt 2016 (1++); Hansen 2017 (1+); Best 2016 (1+); Escamilla-Nunez 2014 (1+); Berman 2016 (1–); Gunaratne 2019 (1++); Lapillonne 2014 (2+); Maslova 2019 (2++); Sordillo 2019 (2++); Yu 2015 (2++); Standl 2014 (2++); Magnusson 2018 (2+); Bisgaard 2016 (1++); Warstedt 2016 (1++); D’Vaz 2012 (1+); Furuhjelm 2011 (1+)
Level of consensus:	Strong consensus

**3. Allergen exposure/allergen-specific immunotherapy. Table10:** 3.1. Pets.

**Level of recommendation**	**Statement**
A/B	**Background:** Several epidemiologic studies have shown that keeping dogs in the first years or the first 3 years of life has a primary protective effect against the development of allergies and asthma. There is still conflicting data with regard to keeping cats or other typical pets.
	**Recommendations:** For families without a recognizably increased risk of allergies, keeping of cats or dogs should not be limited for reasons of primary allergy prevention. (A)
	Families with an increased risk of allergies or with children with pre-existing atopic eczema should not start keeping a cat. (B)
	Families with an increased risk of allergies should not be advised against keeping dogs. (B)
	**Statement:** With regard to pets other than cats and dogs, no recommendations can be made on the primary prevention of allergies and asthma. There is no evidence for giving away existing pets for reasons of allergy prevention.
Level of evidence	Dogs: Marrs 2019 (1–); Collin 2015 (2+); Fall 2015 (2+); Hesselmar 2018 (2–); Al-Tamprouri 2019 (2++)
	Cats: Al-Tamprouri 2019 (2++); Milanzi 2019 (2++)
Level of consensus:	Strong consensus

**3. Allergen exposure/allergen-specific immunotherapy. Table11:** 3.2. Mites.

**Level of recommendation**	**Statement**
A/B	**Background:** Previous studies did not show that reducing the allergen content in the home environment was a reliably effective method for primary allergy prevention. Studies on the correlation between the early exposure to house dust mites, animal dander, and endotoxins and the later development of asthma and/or allergic sensitizations show partially contradicting results.
	**Recommendation:** Interventions to reduce exposure to house dust mite allergens in the home, e.g., by using mite allergen-proof mattress covers (encasings) should not be used with the aim of primary prevention. (B)
	In patients with an existing mite allergy, mite allergen reduction measures should be used, as there is proof of effectiveness. (Tertiary prevention). (A)
Level of evidence	Callesen 2014 (2+); O`Connor 2018 (2++); Lynch 2014 (2+); Karvonen 2014 (2+); Karvonen 2019 (2+); Thorne 2015 (4); Loo 2018 (level of evidence not indicated)
Level of consensus:	Consensus

**3. Allergen exposure/allergen-specific immunotherapy. Table12:** 3.3. Allergen-specific immunotherapy.

**Level of recommendation**	**Statement**
B	**Background:** Some studies have examined the development of allergic sensitization to further/new allergens during the course of AIT, mostly in children with allergic rhinitis/ allergic rhinoconjunctivitis or asthma.
	Two studies were placebo-controlled and were carried out in infants with atopy or in IgE-sensitized, non-allergic children. These studies provided some indication that primary preventive AIT with house dust mite extract can prevent sensitization to other allergens in the first 2 years of life. A modifying effect on allergic symptoms could not be demonstrated.
	**Statement:** AIT for prevention of allergic sensitization and allergic symptoms in infants with increased atopy risk (primary prevention) cannot be recommended at the moment.
	To avoid allergic sensitization to further allergens and allergic symptoms in already sensitized, non-allergic children (secondary prevention), an AIT cannot be recommended at the moment.
	**Recommendation:** In patients with pre-existing allergic rhinitis/rhinoconjunctivitis, AIT to prevent a not yet manifest asthma should be recommended. (Tertiary prevention). (B)
Level of evidence	Crimi 2004 (1–); Marogna 2008 (1–); Szepfalusi 2014 (1+), Zolkipli 2015 (1++); Kristiansen 2018 (1+); Halken 2017 (1+); Jacobsen 2007 (1–); Song 2014 (1+); Valovirta 2017 (1+); Grembiale 2000 (1+)
Level of consensus:	Strong consensus

**4. Biodiversity and other factors. Table13:** 4.1. Biodiversity.

**Level of recommendation**	**Statement**
A/B	**Statement:** There are clear indications that growing up on a farm protects against the development of asthma and allergic diseases.
	This is mediated by early unspecific immune stimulation, among others through the microbial composition of the house dust.
	**Statement:** A recommendation regarding the prevention of atopic diseases through child daycare cannot be given due to the heterogeneous study data.
	**Statement:** There is no proof that vaccinations increase the risk of allergies, but there are indications that vaccinations can lower the risk of allergies.
	**Recommendation:** All children, including children at risk, should be vaccinated according to the current recommendations. (A)
	**Recommendation:** When giving advice on the mode of delivery, it should be taken into account that children who were born through an elective caesarean section have a slightly increased risk of asthma. (B)
Level of evidence	Kirjavainen 2019 (2++); Nicklaus 2019 (2++); Louis 2014 (2++); Brick (2+); Cheng 2014 (2+); Linehan 2014 (2+); Thestesen 2018 (1+); Baxter 2018 (1+); Rusconi 2017 (2+); Kahr 2015 (2+); Wu 2016 (2+); Sevelstedt 2016 (2++); Chu 2017 (2+); Lee 2014 (2+); Brandao 2016 (2+)
Level of consensus:	Strong consensus

**4. Biodiversity and other factors. Table14:** 4.2. Antibiotics and non-steroidal anti-inflammatory drugs.

**Level of recommendation**	**Statement**
	**Statement:** When using *antibiotics in infancy,* it should be taken into account that the use of antibiotics in the first 2 years of life is associated with a moderate increase in the risk of allergic asthma and a slight increase in the risk of hay fever and eczema in later life.
	*Antibiotic use in pregnancy* is associated with a moderate increase in the child’s risk of developing asthma and AD later in life.
	The use of paracetamol and other NSAIDs in toddlers or mothers during pregnancy cannot be clearly associated with an increased risk of asthma and rhinitis. No data on atopic dermatitis are available.
Level of evidence	Antibiotics: Ahmadizar 2018 (1–); Batool 2016 (2–); Goksör 2013 (2+); Hoskin-Parr 2016 (2+); Ong 2014 (2–); Pitter 2016 (2+); Wang 2013 (2++); Yamato-Hanada 2017 (2+); Kashanian 2017 (2-); Stensballe 2013 (2+); Örtqvist 2013 (2++); Stokholm 2014 (2+); Wohl 2015 (2–); Metzler 2019 (2+); Wu 2016 (2++); Metsälä 2015 (2++)
	Studies on analgesics: Amberbier 2014 (2+); Batool 2016 (2–); Cheelo 2015 (2–); Penarando 2015 (2+); Wang 2013 (2++); Hoeke 2016 (2+); Liu 2016 (2+); Sordillo 2015 (2++); Chu 2016 (2–); Magnus 2016 (2++); Piler 2016 (2+)
Level of consensus:	Strong consensus

**4. Biodiversity and other factors. Table15:** 4.3. Skin barrier.

**Level of recommendation**	**Statement**
	**Statement:** It has not been consistently shown that primary prevention in infants with an atopic family history can be achieved by daily moisturizing of healthy skin.
	**Comment:** At the present time, based on the available evidence, no recommendation can be made for daily moisturizing of healthy baby skin with the aim of primary prevention of eczema and allergies – even in families with an increased allergy risk.
	**Recommendation:** In infants and children with visibly dry skin moisturizing creams should be used regularly. (Expert opinion)
Level of evidence	Horimukai 2014 (1++); Simpson 2014 (1+); Chalmers 2020 (1–); Skjerven 2020 (1+); McClanahan 2019 (2+); Dissanayake 2019 (2+)
Level of consensus:	Consensus

**5. Pollutants. Table16:** 5.1. Tobacco smoke.

**Level of recommendation**	**Statement **
A	**Background:** Active and passive exposure to tobacco smoke increase the risk to develop allergies. In particular, the risk of asthma is increased in preschool age and early school age. This already applies during pregnancy. The risk to develop allergies and especially asthma by passive exposure to tobacco smoke during pregnancy is especially high in children with a positive family history for allergies.
	**Recommendation:** Active and passive exposure to tobacco smoke should be avoided. This already applies during pregnancy. (A)
Level of evidence	Hollams 2014 (2+)
Level of consensus:	Strong consensus

**5. Pollutants. Table17:** 5.2. Mold exposure and humidity.

**Level of recommendation**	**Statement**
B	**Recommendation:** An indoor climate that favors the growth of molds (high humidity, poor ventilation) should be avoided. (B)
Level of evidence	Milanzi 2019 (2+); Thacher 2017 (2++); Karvonen 2015 (2+); Wen 2015 (2+)
Level of consensus:	Consensus

**5. Pollutants. Table18:** 5.3. Indoor air pollutants.

**Level of recommendation**	**Statement**
B	**Recommendation:** Exposure to indoor air pollutants should be kept low. (B)
Level of evidence	Madureira 2016(2–); Callesen 2014 (2+); O´Connor 2018 (2++)
Level of consensus:	Strong consensus

**5. Pollutants. Table19:** 5.4. Motor vehicle emissions.

**Level of recommendation**	**Statement**
A	**Background:** Exposure to nitrogen oxides, ozone, and particulate matter with a particle size of < 2.5 µm (PM 2.5) is associated with an increased risk, especially for asthma.
	**Recommendation:** Exposure to vehicle emissions should be kept low. (A)
Level of evidence	Deng 2016 (2+); Brunst 2015 (2++); Gruzieva 2013 (2+); Hasunuma 2016 (2+); Hsu 2015 (2–); Molter 2015 (2++); Nishimura 2013 (2–); Rancière 2017 (2++); Ranzi 2014 (2+); Tétreault 2016 (2+); Kathunia 2016 (not rateable)
Level of consensus:	Strong consensus

**5. Pollutants. Table20:** 5.5. Chlorinated pool water.

**Level of recommendation**	**Statement **
	**Statement:** Regular visits to swimming pools to prevent allergies, rhinitis, and eczema should not be discouraged.
Level of evidence	Andersson 2015 (2+); Font-Ribera 2014 (2+)
Level of consensus:	Strong consensus

**6. Psychosocial factors. Table21:** 

**Level of recommendation**	**Statement**
	**Background:** There are hints that stressful psychosocial factors in the mother during pregnancy and after birth (depression, difficult life events, etc.) can increase the risk of later atopic disease in children up to the age of 14. A high level of social support and high maternal sensitivity, on the other hand, seem to reduce the risk of childhood atopic dermatitis.
	**Statement:** No practical recommendations for action for targeted allergy prevention can currently be given based on available data.
Level of evidence	Andersson 2016 (1++); Alton 2016 (1+); Kozyrskyj 2017 (2++); Wang 2016 (2++); Letourneau 2017 (2++); El-Heis 2017 (2+); Brew 2017 (2+); Guxens 2014 (2++); Hartwig 2014 (2+); Lee 2016 (2+); Larsen 2014 (2+)
Level of consensus:	Consensus
